# Is screening for chronic kidney disease in patients with diabetes mellitus being properly conducted in primary care?

**DOI:** 10.1590/2175-8239-JBN-2021-0210

**Published:** 2022-02-23

**Authors:** Jaquelice Aparecida Lopes, Mariana Caroliny Ferreira, Alba Otoni, André Oliveira Baldoni, Caroline Pereira Domingueti

**Affiliations:** 1 Universidade Federal de São João del-Rei, Campus Centro-Oeste Dona Lindu, Divinópolis, MG, Brasil.

**Keywords:** Renal Insufficiency, Chronic, Diabetes Mellitus, Type 2, Albuminuria, Glomerular Filtration Rate, Diabetic Nephropathies, Insuficiência Renal Crônica, Diabetes Mellitus Tipo 2, Diabetes Mellitus, Albuminúria, Taxa de Filtração Glomerular, Nefropatias diabéticas

## Abstract

**Introduction:**

Screening patients with diabetes mellitus (DM) for chronic kidney disease (CKD) enables early diagnosis and helps to establish adequate treatment and avoid possible damages to health associated with disease progression. This study aimed to verify whether screening for CKD has been properly conducted in populations with diabetes mellitus seen at primary care clinics.

**Methods:**

This descriptive study included 265 individuals with DM seen at Basic Healthcare Clinics in Divinópolis, MG, Brazil. Clinical and laboratory data were collected from the Integrated Health System. Frequency of testing and kidney function evaluations performed within the last 12 months were calculated along with the proportion of patients with increased urinary albumin excretion (UAE) and decreased glomerular filtration rate (GFR) to determine the proportion of patient with kidney involvement.

**Results:**

We found that 41.2% of the patients had kidney involvement and that 61.2% of the individuals with kidney involvement were on nephroprotective medication. Of the 21.9% tested for isolated albuminuria, 46.5% had increased UAE. The albumin-to-creatinine ratio (ACR) was measured in 12.1% of the patients, with 43.8% having an increased ACR. We found that 89.0% of the patients had their serum creatinine levels measured, and that 33.1% had a decreased GFR.

**Conclusion:**

CKD screening was more frequently performed via the GFR than UAE, a parameter analyzed only in a small proportion of patients. Therefore, CKD screening for patients with diabetes is not being performed properly in primary care.

## Resumo

**Introdução:**

O rastreio da doença renal crônica (DRC) em pacientes com diabetes (DM) possibilita o diagnóstico precoce e ajuda a estabelecer um tratamento adequado, evitando possíveis danos à saúde pela progressão da doença. O objetivo deste trabalho foi verificar se o rastreio da DRC está sendo feito de maneira adequada entre diabéticos acompanhados na atenção primária à saúde.

**Métodos:**

Estudo descritivo com 265 pacientes com DM atendidos nas Unidades Básicas de Saúde de Divinópolis, MG. A coleta de dados clínicos e laboratoriais foi realizada por meio de consulta ao Sistema Integrado de Saúde. Foram calculadas a frequência de realização dos exames de avaliação da função renal nos últimos 12 meses e a frequência de pacientes com excreção urinária de albumina (EUA) aumentada e a taxa de filtração glomerular (TFG) reduzida, e assim determinada a frequência de pacientes com comprometimento renal.

**Resultados:**

Foi observado que 41,2% dos pacientes têm comprometimento renal; dentre esses, 61,2% utilizam algum medicamento nefroprotetor. Apenas 21,9% realizaram o exame de albuminúria isolada, dos quais 46,5% apresentaram albuminúria aumentada. O exame de relação albumina/creatinina (RAC) foi realizado por 12,1% dos pacientes, dos quais 43,8% apresentaram RAC aumentada. Foi observado que 89,0% dos pacientes realizaram o exame de creatinina sérica, dos quais 33,1% apresentaram TFG reduzida.

**Conclusão:**

Foi observado maior índice de rastreio da DRC por meio da TFG em relação ao rastreio por meio da EUA, o qual foi realizado por pequeno número de pacientes. Portanto, o rastreio da DRC não está sendo realizado adequadamente na atenção básica ao diabético.

## Introduction

Chronic kidney disease (CKD) results from anomalies in the structure/function of the kidneys and produces health complications that persist for more than three months and lead to high rates of morbidity and mortality^1^. One of its main causes is inadequate management of diabetes mellitus (DM), which leads to microvascular complications and the development of CKD^2^.

CKD may be defined based on decreased glomerular filtration rate (GFR) (< 60 mL/min/1.73m^2^), increased urinary albumin excretion (UAE) [albumin-to-creatinine ratio (ACR) ≥ 30 mg/g or observation of albumin levels ≥ 30 mg in 24-hour urinary protein tests] or abnormal imaging findings. CKD is categorized in three stages based on albuminuria or in five stages based on the GFR, with the last stage of disease corresponding to end-stage renal disease^3^.

Between 30% and 50% of the individuals with DM develop CKD. In addition, DM is the second main cause of CKD in patients with kidney failure^3^. According to the 2019 Brazilian Dialysis Census, the primary cause of CKD was DM for 32% of the individuals on dialysis^4^.

Since patients with early CKD do not develop symptoms, screening must be initiated as soon as a diagnosis of DM is established and repeated annually to allow the early diagnosis of CKD. It is extremely important that all patients with a history of more than five years of DM are screened for CKD. Since a significant proportion of patients with CKD may have normal UAE levels but a decreased GFR, UAE must be assessed jointly with the GFR in the screening for CKD of patients with DM^5^.

Patients with CKD present symptoms only as they move into more advanced stages of the disease, when associated complications such as anemia, metabolic acidosis, malnutrition, and changes in the metabolism of calcium and phosphorus set in, placing them at risk of end-stage renal disease and death^5^.

Therefore, screening patients with DM for CKD is extremely important, since it may help the early establishment of a diagnosis of CKD, thereby decreasing the impact of the disease on society and on the public healthcare system, since patients with the condition may be offered proper treatment with nephroprotective medication to decrease the occurrence of complications and attenuate the cases of patients in need of renal replacement therapy^3^.

Considering the scarcity of observational studies about screening patients with DM for CKD, this study aimed to verify whether the screening for CKD of patients with DM has been properly conducted at Basic Healthcare Clinics in Divinópolis, MG, Brazil.

## Method

This descriptive study was carried out in Divinópolis, MG, Brazil, a municipality with 32 Family Health Teams (FHTs) and 11 Basic Care Teams (BCTs), which see about 10,800 individuals with type 2 DM. The size of the sample (n=241) was calculated based on a population of 10,800 subjects with type 2 DM considering a sampling error of 5% and a confidence level of 95%. An additional 10% were added to account for possible losses, thus bringing the size of the sample to 265 patients.

Two FHTs or BCTs were drawn from each of the six healthcare districts of Divinópolis for purposes of patient data collection. Data from 265 patients was collected. The number of patients selected from each FHT or BCT was proportional to the total number of patients registered with each respective Team. The following inclusion criteria applied: having clinical and laboratory diagnosis of type 2 DM^6^ and having clinical and laboratory data recorded in the Integrated Health System. Exclusion criteria were not defined.

The collection of patient clinical and laboratory data was performed based on searches conducted on a secondary information source, the Integrated Health System. In order to analyze the quality of screening for CKD, we calculated the frequency with which glycated hemoglobin (HbA1c) tests were performed within the last six months and how frequently tests to assess kidney function based on isolated albuminuria, ACR, serum creatinine, and estimated GFR based on the CKD-EPI equation were performed within the last year.

In addition, the proportion of patients with inadequate blood glucose management (HbA1c ≥ 7.0%), moderately increased UAE (isolated albuminuria ≥ 14 and < 174 mg/L or ACR ≥ 30 mg/g and < 300 mg/g), severely increased UAE (isolated albuminuria ≥ 174 mg/L or ACR ≥ 300 mg/g) and decreased GFR (< 60 mL/min/1.73m^2^)^2^ was calculated. The proportion of patients with kidney involvement defined as presence of isolated albuminuria ≥ 14 mg/mL and/or ACR ≥ 300 mg/g and/or GFR < 60 mL/min/1.73m^2^ and the proportion of patients taking nephroprotective medication (angiotensin-converting-enzyme inhibitors or angiotensin II receptor blockers) were also calculated.

The study design was approved by the Ethics Committee for Research with Human Beings of the Federal University of São João del Rei - Midwest Campus (CEP CCO - nº 2.433.578). The use of informed consent terms was not required.

## Results

The patients included in the study were predominantly elderly and most were females. In regard to height, weight, and body mass index (BMI), the study population was overweight^7^. On average, patients were aged 48 years when they were diagnosed with type 2 DM and were living with the disease for 10 years (Table 1).

**Table 1:** Sociodemographic and clinical characteristics of patients with type 2 diabetes mellitus seen at Basic Healthcare Clinics in Divinópolis, MG, Brazil, during the period of one year

Number of patients	265
Males	79 (29.8%)
Females	186 (70.2%)
Age (years)	62 (54-68)
Height (m)	1.63 (1.56-1.70)
Weight (kg)	80 (68-92)
BMI (kg/m2)	29.8 (26.4-32.6)
Age when diagnosed (years)	48 ± 12
Time with type 2 DM (years)	10 (4-20)
Patients on nephroprotective medication	142 (53.6)
Kidney involvement (increased UAE and/or decreased GFR)	98 (41.2)
Patients with kidney involvement on nephroprotective medication	60 (61.2)

Variables presenting a normal distribution were expressed as mean ± standard deviation. Variables not presenting a normal distribution were expressed as median (25%-75% percentile). Categorical variables were expressed as absolute and relative frequencies n (%). Type 2 DM = type 2 diabetes mellitus; UAE = urinary albumin excretion; BMI = body mass index; GFR = glomerular filtration rate.

The data recorded in the Integrated Health System revealed that just over half of the patients were on nephroprotective medication and that 98 (41.2%) had increased UAE and/or a decreased GFR indicative or kidney involvement. More than 60% of the patients with kidney involvement were on nephroprotective medication.

Table 2 shows the laboratory data of patients with type 2 DM. A total of 239 patients (90.2%) were monitored for HbA1c within the last six months, with levels averaging 8.3%. Still in regard to HbA1c, a pattern of poor blood glucose management was found in the study population^3^.

**Table 2:** Laboratory data and frequency with which tests to screen for chronic kidney disease were performed in patients with type 2 diabetes mellitus at Basic Healthcare Clinics in Divinópolis, MG, Brazil

HbA1c (%)	8.3 ± 2.1
HbA1c ≥ 7.0% (n, %)	146 (61.1)
HbA1c test performed within the last six months	239 (90.2)
Isolated albuminuria (mg/L)	5.3 (1.4 - 43.0)
Isolated albuminuria ≥ 14 and < 174 mg/L (n, %)	22 (37.9)
Isolated albuminuria ≥ 174 mg/L (n, %)	5 (8.6)
Isolated albuminuria test performed within the last year	58 (21.9)
ACR (mg/g)	11.2 (5.9 - 288.0)
ACR ≥ 30 mg/g and < 300 mg/g (n, %)	6 (18.8)
ACR ≥ 300 mg/g (n, %)	8 (25.0)
ACR test performed within the last year	32 (12.1)
Serum creatinine (mg/dL)	1.0 (0.9 - 1.3)
Serum creatinine test performed within the last year	236 (89.0)
GFR (mL/min/1.73m2)	67 ± 17
GFR < 60 mL/min/1.73m2 (n, %)	78 (33.1)
GFR test performed within the last year	236 (89.0)
Isolated albuminuria ≥ 14 mg/mL and/or ACR ≥ 30 mg/g and/or GFR < 60 mL/min/1.73m2	98 (41.2)
Isolated albuminuria and/or ACR and/or GFR test performed within the last year	238 (89.8)
UAE (isolated albuminuria or ACR) and GFR test performed within the last year	57 (21.5)

Variables presenting a normal distribution were expressed as mean ± standard deviation. Variables not presenting a normal distribution were expressed as median (25%-75% percentile). Categorical variables were expressed as absolute and relative frequencies n (%). UAE = urinary albumin excretion; HbA1c = glycated hemoglobin; ACR = albumin-to-creatinine- ratio; GFR = glomerular filtration rate.

Only 58 patients (21.9%) were tested for isolated albuminuria within the last year, and a median level of 5.3 mg/L was found. About half of these patients had moderately or severely increased UAE^6^. In addition, we found that only eight patients had been tested for isolated albuminuria more than once within the last year.

A total of 32 patients (12.1%) had the ACR estimated within the last year, and a median ratio of 11.2 mg/g was found. Some of these patients had moderately or severely increased UAE^1^. Only four patients had their ACR estimated within the last year.

Within the last year, 236 patients (89%) were tested for serum creatinine, from which the GFR may be estimated. The median serum creatinine level was 1.0 mg/dL and the mean GFR was 67 mL/min/1.73m^2^. A considerable proportion of these patients had a GFR below 60 mL/min/1.73m^2^ indicative of kidney involvement^1^. No significant difference was found between healthcare districts in terms of screening for CKD based on UAE or GFR.

Considering the three parameters used to screen patients for CKD (isolated albuminuria, ACR, and GFR), we found that 236 patients (89.8%) were assessed for at least one of these parameters within the last year and that only 57 patients (21.5%) were tested for UAE (isolated albuminuria or ACR) and had the GFR estimated within the last year. Our results indicated that 98 patients (41.2%) had isolated albuminuria ≥ 14 mg/mL and/or ACR ≥ 30 mg/g and/or GFR < 60 mL/min/1.73m^2^ indicative of kidney involvement^6^.

## Discussion

Tests performed with the purpose of screening patients with type 2 DM for CKD were conducted for a significant portion (89.8%) of the patients included in the study. Screening based on the GFR was performed more often than methods based on isolated albuminuria or ACR. This is possibly due to the fact that the GFR is considered one of the best parameters to assess kidney function in patients with or without DM, since it reflects renal excretion function and is more closely correlated with clinical outcomes than blood creatinine, which should not be used in isolation^1^.

Studies have described different groups of patients with CKD. One of these groups comprises individuals with a decreased GFR without albuminuria^8^. Interestingly, some patients have increased UAE levels and a normal GFR^3^. The GFR alone is not enough to establish a diagnosis of early CKD and albuminuria is an important predictor of risk of developing CKD; therefore, screening patients based on both parameters is a more effective means to identify cases of early CKD^9^. This is why UAE and the GFR have been included in the guidelines issued by the Brazilian Society for Diabetes to screen patients for CKD^3^.

A small portion of patients was tested for isolated albuminuria within the last year, and even fewer had the ACR calculated. This finding differed from the results published in other studies about the prevalence of kidney disease in different regions, in which the assessment of kidney function via the ACR prevailed^10,11^. The KDIGO recommends that the ACR should be preferred over isolated albuminuria on account of the interferences introduced by the dilution of urine samples in isolated albuminuria testing, while in the ACR creatinine and albumin are diluted by the same proportion, which thus normalizes results^1^. This finding may be explained by the recommendation stated in the Guidelines of the Brazilian Society for Diabetes, in which isolated albuminuria testing is preferred due to its low cost and good accuracy^3^. Furthermore, a study and meta-analysis showed that there is no significant difference between the two methods at detecting moderately increased albuminuria^12^.

Kidney involvement was seen in 41.2% of the patients (patients with isolated albuminuria ≥ 14 mg/mL and/or ACR ≥ 30 mg/g and/or GFR < 60 mL/min/1.73m^2^). Poor blood glucose management (HbA1c ≥ 7.0%) observed in a portion of the study population, alongside many other factors, may explain this finding, since chronic exposure to hyperglycemia is deemed an important factor in the onset and progression of vascular complications such as diabetic kidney disease (DKD)^13^. Additionally, the development of DKD has also been correlated with factors such as advanced age, long-standing DM, and overweight/obesity. Patients included in the study were aged 62 years on average, had type 2 DM for 10 years, and a BMI indicative of overweight^11^. It is important to stress that the incidence of DKD might have been even higher if the ACR was calculated or isolated albuminuria tests were performed more often in patients, since some might present increases in isolated albuminuria or ARC despite having a normal GFR^3^.

In regard to the estimation of isolated albuminuria, 46.5% of the tested patients had diagnostic values consistent with DKD (≥ 14 mg/L). In terms of the ACR, a parameter calculated less often, 43.8% of the tested patients had increased albuminuria^1^. Our findings were similar to the data published by Alves et al. (2017)^10^, in a study that found a considerable number of patients with an ACR moderately or severely increased, in which increased risk of UAE in patients with type 2 DM may be found. Albuminuria assessment is important in the diagnosis of DKD. Its levels should be estimated more often in the study population, since early detection and initiation of treatment for CKD may impede the progression of kidney disease and the introduction of renal replacement therapy^9^.

A study by Vanelli et al. (2018)^14^ designed to screen patients for CKD based on the Screening for Occult Renal Disease (SCORED) tool found that albuminuria was present in a small number of individuals, suggesting that albuminuria testing has been underused in primary care to screen patients for CKD. Dumont et al. (2021)^15^ looked into the charts of 177 patients seen at a dialysis center and found that 20.3% had been diagnosed with CKD on the day they were started on renal replacement therapy. Given the asymptomatic progression of CKD, it is essential to screen populations at risk for the disease, such as individuals with type 2 DM.

Serum creatinine in isolation should not be used to screen patients for CKD on account of its low diagnostic sensitivity and specificity for DKD. Its relatively frequent use within the last year is explained by the use of this marker in the estimation of the GFR. Median serum creatinine is one of the parameters established in the 2012 KDIGO guidelines. However, a study about the prevalence of chronic kidney disease in the Brazilian Southeast found some patients with acceptable serum creatinine levels and an altered GFR. This was also observed in our study. Median serum creatinine was 1.0 mg/mL and 33.1% of the patients had a decreased GFR, which illustrates the higher sensitivity of the GFR in the early diagnosis of DKD^10^.

The considerable prevalence of use of nephroprotective medication among these patients (53.6%) further supports treatment strategies adopted for patients with DKD to mitigate progression of advanced disease^3,16^. Despite the significant prevalence of use of nephroprotective medication, about 40% of the patients with kidney involvement were not on drugs of this class. A study enrolling 177 patients from a dialysis center found that 63.2% of the patients did not undergo conservative therapy with nephroprotective medication^15^. Nephroprotective drugs promote the vasodilation of afferent arterioles, thereby decreasing glomerular filtration and glomerular filtration pressure and introducing a nephroprotective effect in the long term. The use of nephroprotective drugs has been associated with delayed development of kidney disease and normalization of UAE in some patients. In this sense, healthcare teams should prescribe drug therapy to patients with kidney involvement in a timely manner to minimize progression into advanced CKD^17,18^.

When all patients - with and without kidney involvement - were considered, we found a high frequency of use of nephroprotective medication. This was not the case in a study that assessed the prevalence of kidney disease in a municipality in Southeastern Brazil, in which none of the patients with DM alone was on nephroprotective medication. However, this same study reported that a good portion of the population with DM and hypertension was on nephroprotective drugs. The significant number of patients without kidney involvement who were on nephroprotective agents may be explained by the possibility of this population having type 2 DM and hypertension^10^.

In reference to patient clinical data, females accounted for most of the included population, as also described in other studies. This may be explained by the fact that women seek medical care more often then men^10^. Included patients had DM for 10 years on average, a disease known for producing vascular complications such as DKD^13^. Age is also a risk factor for the development of CKD on account of the gradual decrease of the GFR with aging^11^. The results observed in regard to weight, height, and the BMI indicated that this was a population suffering from overweight. An increased BMI has been associated with increased risk of developing type 2 DM and microvascular complications^19^. A link has also been shown between weight loss and good blood glucose management, which in turn may prevent the onset of complications in patients with type 2 DM^3^.

A large portion of the patients included in the study had blood glucose levels managed via HbA1c testing. This finding may be explained by the fact that HbA1c level measurement is recommended in the guidelines of the Brazilian Society for Diabetes as a means to manage the blood glucose level of patients with type 2 DM. Our results indicate a need to improve the blood glucose profile of the study population, since the Brazilian Society for Diabetes stipulates that HbA1c levels below 7% help delay the progression of albuminuria in patients with type 2 DM and that hyperglycemia is a risk factor for the development of DKD and other chronic complications associated with DM^3^.

A limitation of this study is that the included population was seen at only one municipality, which means that our findings cannot be extrapolated to describe the status of these matters in Brazil. However, the sex, ethnicity, and age distribution of the population living in Divinópolis, MG, is similar to the one observed in Brazil. Another limitation of our study is that conducting one single test to check for isolated albuminuria or calculate the ACR may yield false-negative and false-positive results on account of the physiological differences between individuals^11^. Lastly, at least two altered results for UAE or the GFR within three to six months are needed to diagnose individuals with CKD^1^.

## Conclusion

The results described in this study showed that although prevalent, CKD has been disregarded in the care of patients with type 2 DM. A greater proportion of patients were screened for CKD based on the GFR when compared to UAE, a parameter considered in a few patients only. However, diabetic patients are not being properly screened for CKD in primary care clinics, since both parameters should be assessed annually. The study also found patients with kidney involvement without a prescription of nephroprotective medication. These findings call for action in public healthcare to improve the screening for CKD of patients with type 2 DM and prescribe drug therapy to patients with kidney function involvement.
